# Ataxic Hemiparesis after Corona Radiata Infarct: Diffusion Tensor Imaging Correlation of Corticoponto-cerebellar Tract Injury

**DOI:** 10.1515/tnsci-2020-0001

**Published:** 2020-01-31

**Authors:** Jun Young Kim, Jeong Pyo Seo, Min Cheol Chang

**Affiliations:** 1 Department of Physical Medicine and Rehabilitation, College of Medicine, Yeungnam University 317-1, Daemyungdong, Namku, Taegu, 705-717, Republic of Korea

**Keywords:** ataxia, corona radiata, cerebral infarct, diffusion tensor tractography, cortico-ponto-cerebellar tract

## To the editor

**Fig 1:**
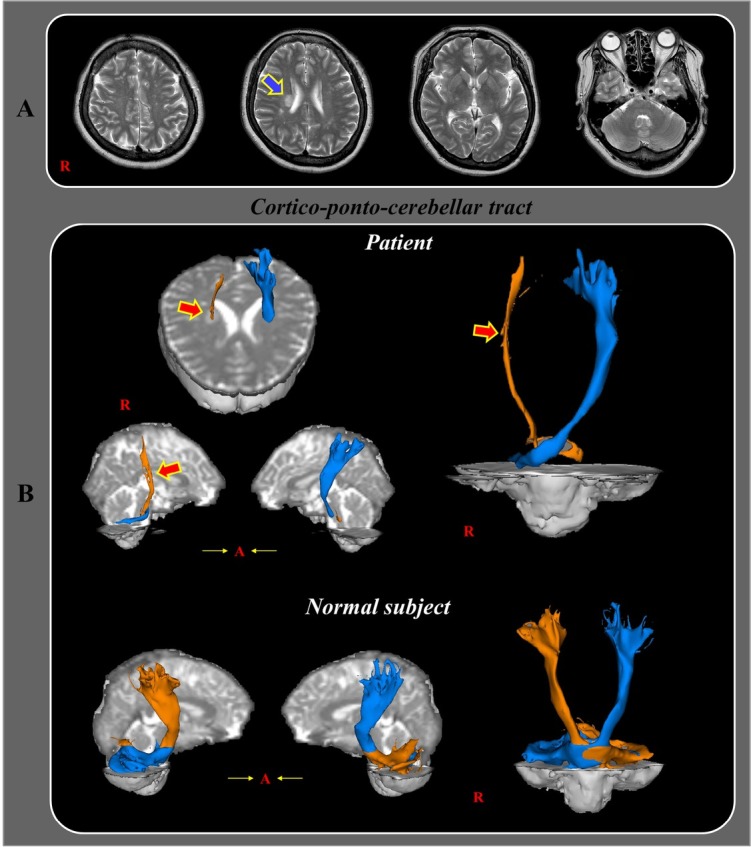
(A) T2-weighted brain magnetic resonance images taken 2 weeks after disease onset showing a right corona radiata infarct (blue arrow). (B) Diffusion tensor tractography in the cortico-ponto-cerebellar tract (CPCT). Thinning of the right CPCT is visible (red arrows). The left CPCT is well-preserved. The bilateral CPCT images in a healthy control (50-year-old male) without any injury are presented.

A 50-year-old female patient underwent conservative management for a right corona radiata infarct at the Department of Neurology in a university hospital (Figure 1A). She had no history of diabetes, hypertension, or heart disease. The patient received a loading dose of clopidogrel (300 mg/day), followed by 75 mg/day plus 100 mg/day aspirin.

She was transferred to the rehabilitation department of the same hospital 2 weeks after infarct onset. Prior to this presentation, the patient had no history of any neurological or psychological disorders. Physical examination revealed ataxia on her left upper and lower limbs. She scored 10 on the Scale for Assessment and Rating of Ataxia (range, 0–40 points with a higher score indicating a poorer state). In addition, she had mild weakness in the left upper and lower limbs (manual muscle test revealed a good grade on the upper and lower limbs). She was able to walk independently indoors with supervision and needed no physical assistance. There were no observations indicating spasticity, sensory deficits, visual disturbance, language impairment (such as dysarthria and aphasia), and dysphasia. Furthermore, she scored all 30 points on the mini-mental state examination. Her thyroid function test results were unremarkable. In addition, the white blood cell count (5,504 μl) and erythrocyte sedimentation rate (20 mm/H) were within normal range.

Diffusion tensor imaging was performed 2 weeks after disease onset using a 6-channel head coil on a 1.5-T Philips Gyroscan Intera (Philips, Ltd, Best, The Netherlands) system with single-shot echo-planar imaging. Seventy contiguous slices were acquired parallel to the anterior commissure-posterior commissure line for each of the 32 non-collinear diffusion sensitizing gradients. The imaging parameters were: acquisition matrix = 96 × 96; reconstructed matrix = 192 × 192; field of view = 240 × 240 mm^2^; repetition time = 10,398 ms; echo time = 72 ms; parallel imaging reduction factor = 2; echo-planar imaging factor = 59; b = 1000 s/mm^2^; and slice thickness = 2.5 mm. Eddy current correction was applied to correct the head motion effect and image distortion before fiber tracking using the Oxford Centre for Functional Magnetic Resonance Imaging of the Brain (FMRIB) Diffusion Software.

Fiber tracking was performed via probabilistic tractography using the default tractography option in the FMRIB Diffusion Software (streamline samples = 5000, step lengths = 0.5 mm, curvature thresholds = 0.2). To reconstruct the cortico-ponto-cerebellar tract (CPCT), a seed region of interest (ROI) was defined at the primary sensorimotor cortex on the axial image. Next, two target ROIs were placed at the anterior portion of pons on the axial image and the contralateral middle cerebellar peduncle on the coronal image [1]. We observed a thinned right CPCT (partial injury) and preserved left CPCT in our patient (Figure 1B).

The cerebellum plays a role in the coordination of voluntary movements and correction of errors in muscle contractions during active movements [2]. Furthermore, it is responsible for timely muscle activation to ensure smooth body movements [2]. Therefore, lesions in the cerebellum and its pathways can result in coordination losses and errors in the timing of muscle activations, which manifest as ataxia, tremor, dysmetria, or nystagmus. The CPCT is a main cerebellar-related neural pathway [2, 3] that arises from the cerebral cortex and enters the pons *via* the corona radiata and internal capsule. It establishes synapses within the pontine nuclei and arrives at the contralateral cerebellar cortex *via* the middle cerebellar peduncle [2]. Our patient’s ataxia, which was not present before the cerebral infarct, manifested in the absence of the main symptoms of Parkinson’s disease, such as rigidity and bradykinesia. In addition, the patient’s thyroid function was normal; therefore, we postulate that the ataxia in the left limbs was caused by injury in the right CPCT (connecting the right cerebral hemisphere and the left cerebellum) at the right corona radiata. In 2004, Arboix et al. reported 35 patients with ataxic hemiparesis among 2500 acute stroke patients over a 12-year period [4]. They showed that corona radiata, internal capsule, or pontine infarcts were significantly associated with ataxic hemiparesis. In addition, 45% of 35 patients were symptom free at the time of discharge. In 2007, Hiraga et al. reported that ataxic hemiparesis is mainly seen in patients with corona radiata, internal capsule, or pontine infarcts [5]. They believed that the CPCT injury caused hemiparetic ataxia after corona radiata, internal capsule, or pontine infarcts without the depiction of neural tracts. In 2015, Marek et al. demonstrated dentato-rubro-thalamo-cortical tract injury in six patients with ataxia after infarct or intracerebral hemorrhage on the pontine, thalamus, and subcortical white matter using diffusion tensor tractography (DTT) [6]. To the best of our knowledge, this is the first study using DTT to demonstrate that CPCT damage can be attributed to the development of ataxia after a corona radiate cerebral infarct. However, our study is limited because we did not compare our DTT results with a patient with a corona radiata infarct who did not exhibit ataxia.

The present study was supported by a National Research Foundation of Korea grant funded by the Korean government (grant no. NRF-2019R1F1A1061348).

## References

[1] Hong JH, Jang SH (2011). Functional magnetic resonance imaging and diffusion tensor tractography of the corticopontocerebellar tract in the human brain. Neural Regen Res.

[2] Choi SM (2016). Movement Disorders Following Cerebrovascular Lesions in Cerebellar Circuits. J Mov Disord.

[3] Jang SH, Chang CH, Jung YJ, Kwon HG (2016). Severe ataxia due to injuries of neural tract detected by diffusion tensor tractography in a patient with pontine hemorrhage: a case report. Medicine (Baltimore).

[4] Arboix A, Bell Y, García-Eroles L, Massons J, Comes E, Balcells M (2004). Clinical study of 35 patients with dysarthria-clumsy hand syndrome. J Neurol Neurosurg Psychiatry.

[5] Hiraga A, Uzawa A, Kamitsukasa I (2007). Diffusion weighted imaging in ataxic hemiparesis. J Neurol Neurosurg Psychiatry.

[6] Marek M, Paus S, Allert N, Mädler B, Klockgether T, Urbach H (2015). Ataxia and tremor due to lesions involving cerebellar projection pathways: a DTI tractographic study in six patients. J Neurol.

